# Excess mortality due to Covid-19? A comparison of total mortality in 2020 with total mortality in 2016 to 2019 in Germany, Sweden and Spain

**DOI:** 10.1371/journal.pone.0255540

**Published:** 2021-08-03

**Authors:** Bernd Kowall, Fabian Standl, Florian Oesterling, Bastian Brune, Marcus Brinkmann, Marcel Dudda, Peter Pflaumer, Karl-Heinz Jöckel, Andreas Stang

**Affiliations:** 1 Institute for Medical Informatics, Biometry and Epidemiology, University Hospital Essen, Essen, Germany; 2 Cancer Registry of North Rhine-Westphalia, Bochum, Germany; 3 Medical Emergency Service of the City of Essen, Essen, Germany; 4 Department for Trauma, Hand and Reconstructive Surgery, University Hospital of Essen, Essen, Germany; 5 Center for Clinical Trials, University Hospital Essen, Essen, Germany; 6 Faculty of Statistics, Technical University of Dortmund, Dortmund, Germany; 7 Department of Epidemiology, School of Public Health, Boston University, Boston, Massachusetts, United States of America; University Magna Graecia of Catanzaro, ITALY

## Abstract

**Introduction:**

Excess mortality is a suitable indicator of health consequences of COVID-19 because death from any cause is clearly defined contrary to death from Covid-19. We compared the overall mortality in 2020 with the overall mortality in 2016 to 2019 in Germany, Sweden and Spain. Contrary to other studies, we also took the demographic development between 2016 and 2020 and increasing life expectancy into account.

**Methods:**

Using death and population figures from the EUROSTAT database, we estimated weekly and cumulative Standardized Mortality Ratios (SMR) with 95% confidence intervals (CI) for the year 2020. We applied two approaches to calculate weekly numbers of death expected in 2020: first, we used mean weekly mortality rates from 2016 to 2019 as expected mortality rates for 2020, and, second, to consider increasing life expectancy, we calculated expected mortality rates for 2020 by extrapolation from mortality rates from 2016 to 2019.

**Results:**

In the first approach, the cumulative SMRs show that in Germany and Sweden there was no or little excess mortality in 2020 (SMR = 0.976 (95% CI: 0.974–0.978), and 1.030 (1.023–1.036), respectively), while in Spain the excess mortality was 14.8% (1.148 (1.144–1.151)). In the second approach, the corresponding SMRs for Germany and Sweden increased to 1.009 (1.007–1.011) and 1.083 (1.076–1.090), respectively, whereas results for Spain were virtually unchanged.

**Conclusion:**

In 2020, there was barely any excess mortality in Germany for both approaches. In Sweden, excess mortality was 3% without, and 8% with consideration of increasing life expectancy.

## Introduction

In the Corona pandemic, the Swedish special path, which is by no means free of restrictive measures, but far less restrictive than pandemic control in other European countries, has triggered strong controversy [1]. While critics point to the high number of COVID-19-associated deaths in Sweden during the first wave, others consider the Swedish path as a model for other countries. The aim of the present study was to investigate the excess mortality in Sweden, Germany and—as one of the countries with the highest COVID-19 associated mortality rates—Spain. This approach, which takes into account deaths from any cause, has the advantage of avoiding problems arising from the considerable variation in definitions of what constitutes a COVID-19 death between different countries [2].

Especially in official statistics, excess mortality is often assessed from numbers of deaths alone [3, 4]. The European Commission publishes data on excess mortality calculated as the percentage of additional mortality in 2020 compared to the baseline period 2016 to 2019. However, this approach does not take demographic changes between 2016 and 2020 into account (e.g., a 20% increase in the number of persons aged 80 and over in Germany), and it does not consider the increase in life expectancy.

Our aim is to compare the overall mortality in 2020 with the overall mortality in 2016 to 2019 in Germany, Sweden and Spain. We take the ageing of these societies and increasing life expectancy into account. Moreover, we estimate not only calendar-week specific standardized mortality ratios (SMRs), but also cumulative SMRs.

## Methods

### Data sources

The death and population figures used for the analyses were extracted from the EUROSTAT database “Population (Demography, Migration And Projections)” [5]. From this database, we extracted the number of death cases from the subset demo_r_mwk_20 by country, year (2016 to 2020), calendar week, and 20-year age groups (< 20, 20–39, 40–59, 60–79, ≥ 80 years). Moreover, we extracted the population size (January 1^st^) of each country by year and the same 20-year age groups from the subset demo_pjangroup. Mid-year population sizes were calculated as the mean of the population sizes on January 1^st^ of the preceding and the following year.

### Statistical analyses

The expected number of deaths in each calendar week of 2020 was calculated for the age groups 0–19, 20–39, 40–59, 60–79 and 80+ from age-group-specific mortality data and population numbers from 2016 to 2019 using the following procedure [6]. First, we calculated age-specific weekly mortality rates throughout the years 2016 to 2019 by dividing the age-specific weekly numbers of deaths by the respective age-specific mid-year population of the country. Then, we calculated mean mortality rates for the years 2016 to 2019 separately for each age group and each calendar week. Finally, we estimated the expected number of deaths in each calendar week of 2020 by multiplying the age-specific weekly mean mortality rates with the age-specific population of the year 2020. As age-specific mid-year populations are not yet available for 2020, we used the age-specific populations for 31^st^ December 2019 instead.

Subsequently, the Standardized Mortality Ratio (SMR) with 95% confidence interval was estimated for each calendar week of 2020 for the total population, by dividing the number of all deaths observed in that calendar week by the number of all deaths expected in that calendar week. In addition to the calendar week-specific SMRs, cumulative SMRs were also estimated: for example, to estimate the SMR cumulatively for calendar week 30, the number of deaths observed in calendar weeks 1 to 30 is divided by the number of deaths expected in those calendar weeks.

#### Sensitivity analysis

Life expectancy continues to improve in European countries. All over Europe, it was 78.1, 78.3, 78.4 and 78.6 years in the years 2016 to 2019 [7]. This is not only due to a decline of child mortality because improvements are seen in all age groups. To take increasing life expectancy into account, we calculated the expected weekly number of deaths by using extrapolated mortality rates instead of mean mortality rates for 2016 to 2019. We extrapolated age-specific weekly mortality rates for 2020 from mortality rates for 2016 to 2019 using an exponential model: d_x_(t) = exp (a_x_ + b_x_(t)) with d_x_: mortality rate of age group x, and t: calendar year.

As a measure of increasing life expectancy, we additionally calculated “relative mortality change” according to the following formula:

$$relative\;mortality\;change=\frac{D2020,s}{(D2016+D2017,s+D2018,s+2019,s)/4}$$ (1)

D2016 is the number of deaths in 2016. D2017s, D2018s, D2019s and D2020s is the standardized number of deaths which would have occurred if the population size and age structure had been the ones of 2016. D2020s also refers to the standard population of 2016, and it is calculated using the extrapolated age-specific weekly mortality rates for 2020 (see above). If age-specific mortality rates actually decrease due to increasing life expectancy, relative mortality change will take values below 1.

## Results

**Fig 1:**
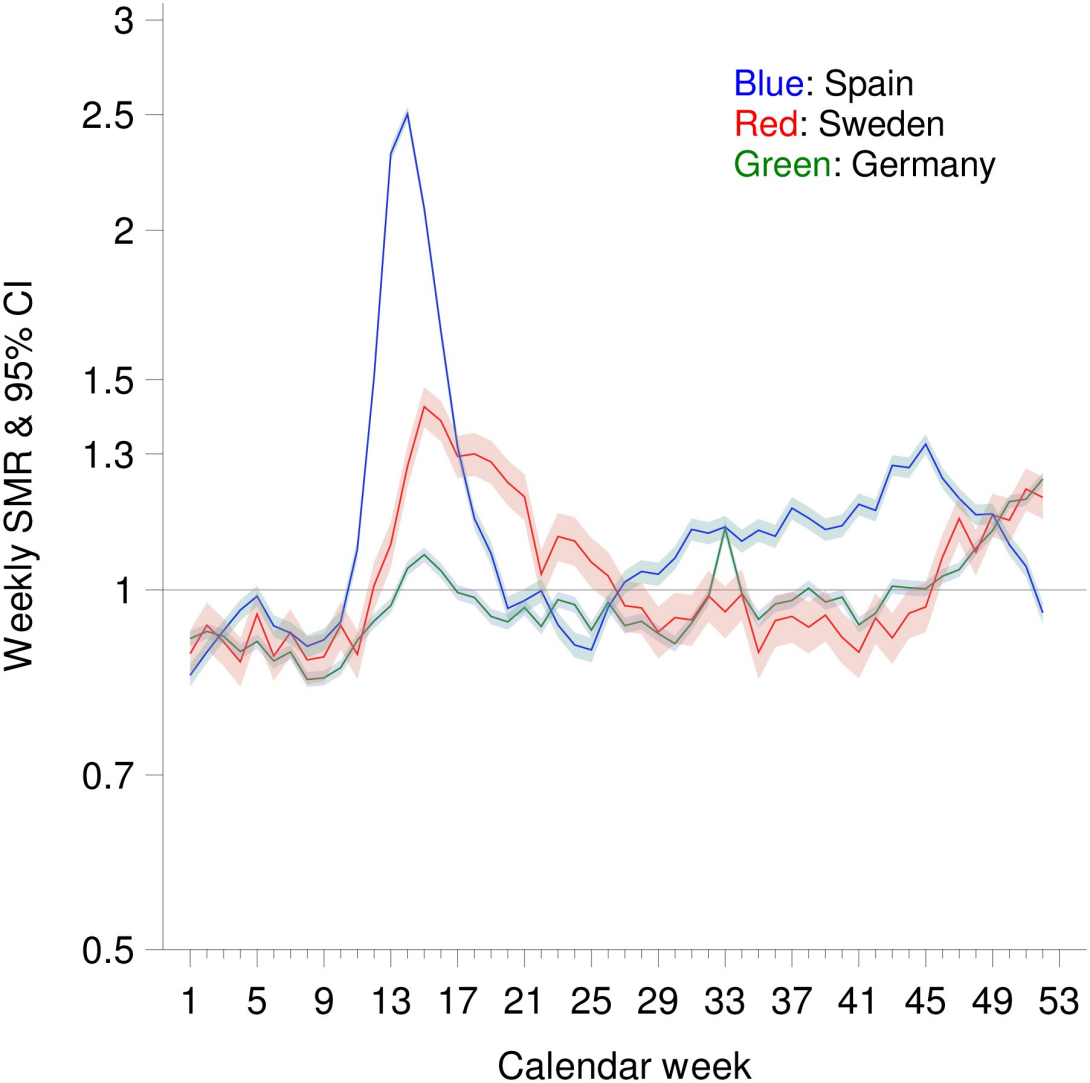
Weekly standardized mortality ratio (SMR) by calendar week in Spain, Germany and Sweden (no consideration of life expectancy).

Fig 1 shows that weekly SMRs in all three countries were below 1 before the onset of the first wave of the Corona pandemic, which began with calendar week 10, so total mortality in 2020 by then was lower than the average of the previous four years. During the first wave, there was high excess mortality in Sweden and especially in Spain. In Germany, on the other hand, there was no excess mortality during the whole period of calendar weeks 10 to 23. The second wave started as early as calendar week 25 in Spain, where it was accompanied by a continuous increase in the calendar-week-specific SMR to 1.324 (95% CI: 1.299–1.350) in calendar week 45, and then decreased to 0.957 (0.937–0.977) by the end of the year. In Sweden and Germany, week-specific SMR values were consistently above 1 after calendar week 46. Weekly SMRs in calendar week 52 were 1.238 (1.223–1.254) in Germany, and 1.195 (1.147–1.243) in Sweden.

**Fig 2:**
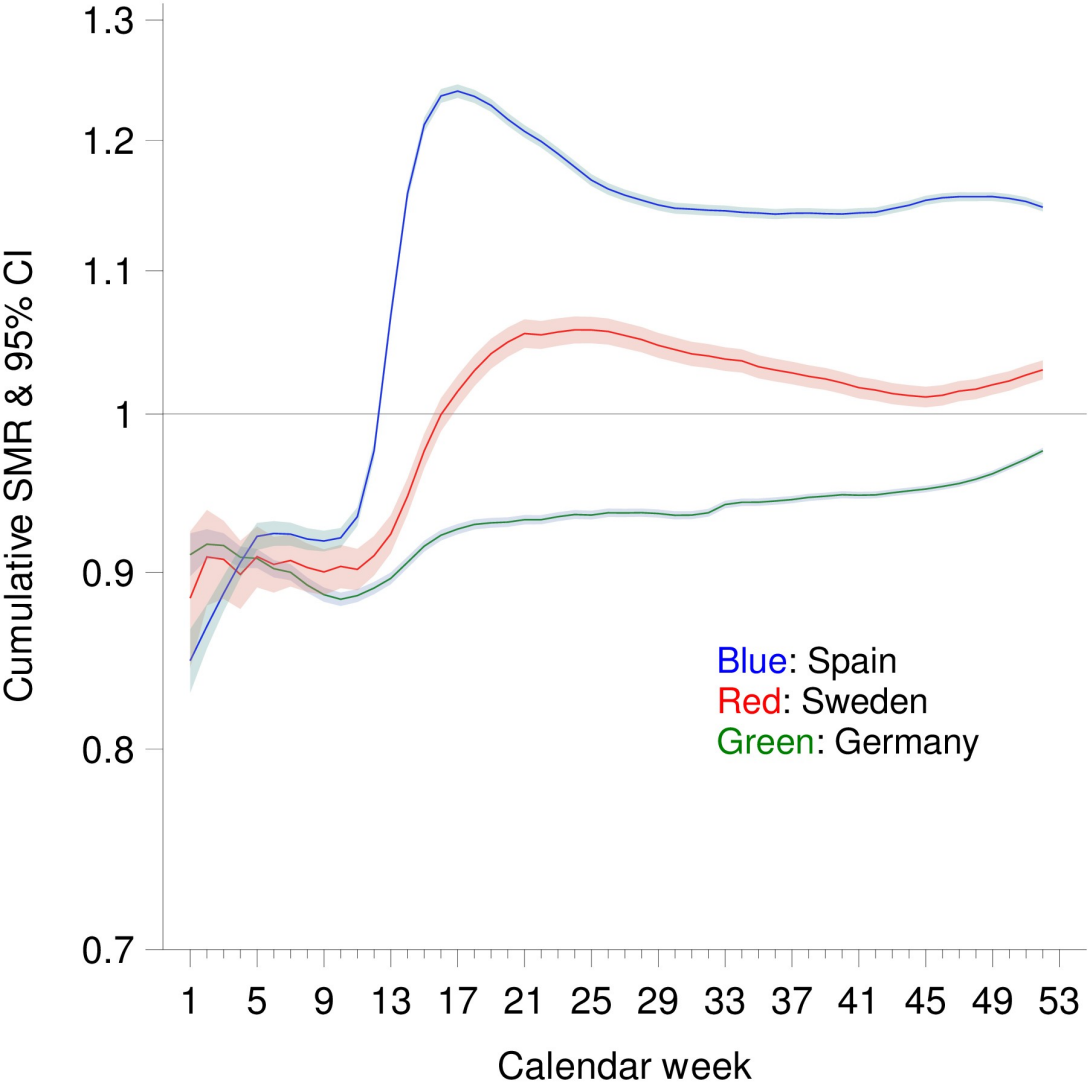
Cumulative standardized mortality ratio (SMR) by calendar week in Spain, Germany and Sweden (no consideration of life expectancy).

At the end of the year, the cumulative SMR in Germany was 0.976 (0.974–0.978) (Fig 2). In Sweden, the cumulative SMR decreased until calendar week 45, and then increased again, and it was 1.030 (1.023–1.036) in calendar week 52. In Spain, the cumulative SMR was 1.148 (1.144–1.151) by the end of the year.

**Fig 3:**
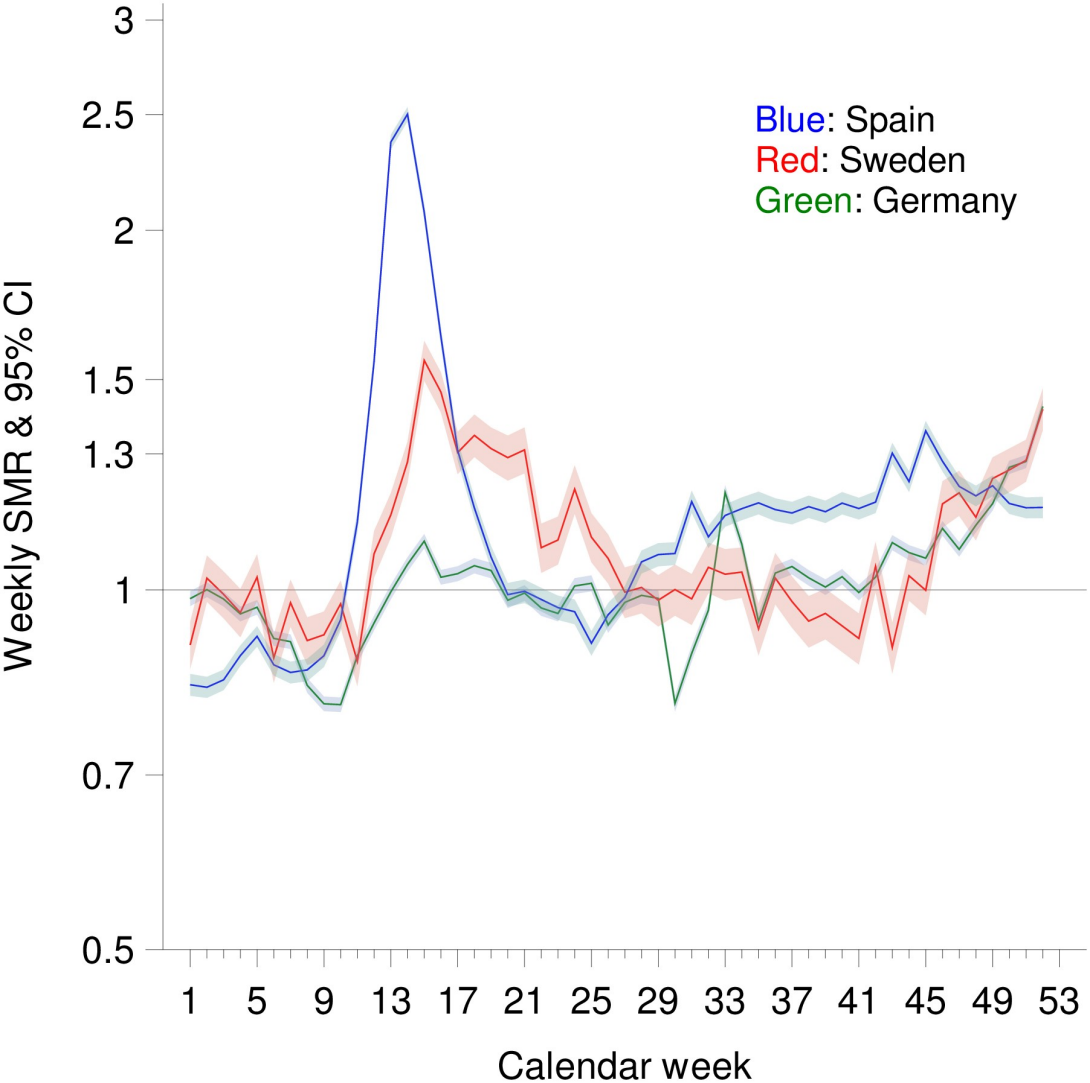
Weekly standardized mortality ratio (SMR) by calendar week in Spain, Germany and Sweden (with consideration of life expectancy).

**Fig 4:**
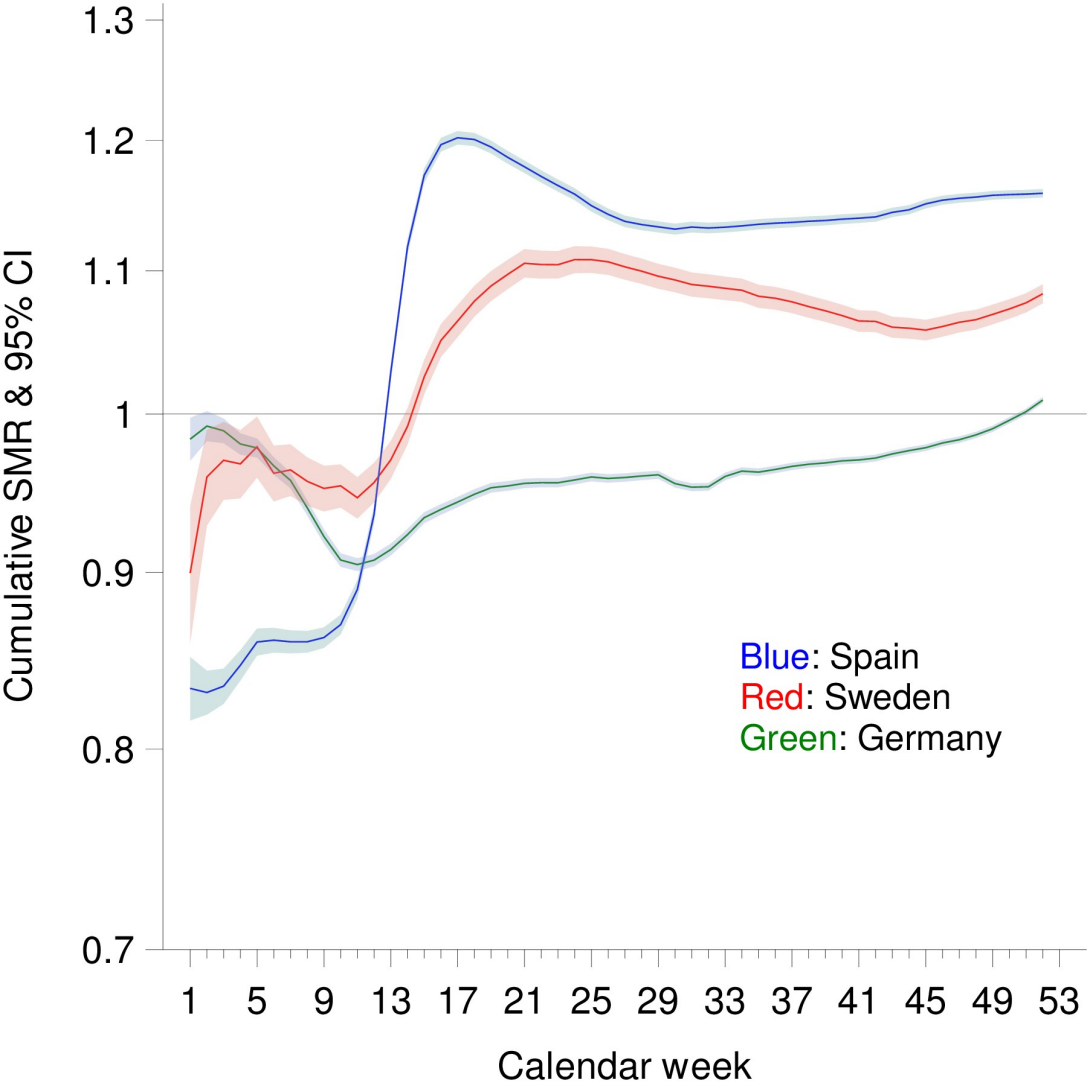
Cumulative standardized mortality ratio (SMR) by calendar week in Spain, Germany and Sweden (with consideration of life expectancy).

Figs 3 and 4 show the weekly and the cumulative SMR values after taking increasing life expectancy into account. Using this approach, cumulative SMR values were higher in all three countries. By the end of the year, the cumulative SMR with consideration of the increase in life expectancy was 1.158 (1.155–1.161) in Spain. In Germany and Sweden, cumulative SMR values by the end of the year were 1.009 (1.007–1.011), and 1.083 (1.076–1.090), respectively, when increase of life expectancy was considered.

Relative mortality change as defined in (1) was 0.991 in Spain, 0.967 in Germany, and 0.951 in Sweden.

## Discussion

The cumulative SMR values which take the demographic development during the years 2016 to 2020 into account show that in Germany overall mortality was lower in 2020 than in the four preceding years, whereas in Sweden excess mortality amounted to 3%. In Spain excess mortality was 15% in 2020.

In the EuroMOMO network, for the time of the first wave of the pandemic excess mortality was observed in Sweden and Spain, but not in Germany (Berlin and Hesse federal states) [4]. For Sweden, excess mortality was found for all age groups 60 years and older for the first wave, but not any more for June and onwards until calendar week 33 [8]. However, in these studies, changes in the age structure of the populations had not been accounted for.

In Germany, cumulative mortality by the end of 2020 was relatively 2.4% lower than in the average of the preceding four years. Earlier studies reported no elevated SMR for January to June (1.00, 95% CI: 0.97–1.04) and an increased SMR (1.03; 95% CI: 1.03–1.04) for the weeks 10–23 [6, 9]. However, these analyses were based on net numbers of death. When the demographic development was taken in account according to the procedure in our main analysis, an SMR of 0.98 (95% CI 0.98–0.99) had been estimated for calendar weeks 10–23 what is in line with our results [6]. The cumulative SMR below 1 observed for Germany does not mean that there were no deaths caused by COVID-19. The SMR values depend strongly on the extent of overall mortality in the preceding years and higher values could be assumed if, for example, there had been fewer influenza deaths in the comparison years. Side effects of pandemic control may lead to both an increase and a decrease in mortality. On the one hand, as an example, in Germany the number of persons who died in traffic accidents was reduced by 18% during March to June 2020 compared to March to June 2019 [10]. On the other hand, in Germany a decline in hospital admissions was reported for cancer and heart failure [11, 12].

Comparing observed mortality in 2020 to mortality in 2016 to 2019, SMR of 1 could be misleading if there was an increase in life expectancy in 2016 to 2019. Assuming that life expectancy had continued to increase in 2020, SMR of 1 could indicate that further gain in life expectancy was nullified by Covid-19. In the sensitivity analyses which were adjusted for continuous decrease of mortality rates and thus took increasing life expectancy into account, cumulative SMRs for Spain increased only slightly, whereas the increase of cumulative SMR values was larger for Germany and Sweden. Even considering increasing life expectancy, the cumulative SMR was only slightly above 1 in Germany, whereas it increased considerably in Sweden from 1.03 (1.02–1.04) to 1.08 (1.08–1.09). Relative mortality change as defined in the Methods section was below 1 in all three countries, and thus indicated a decrease of mortality rates in Germany, Sweden and Spain during 2016 to 2019. This decrease was smallest in Spain and largest in Sweden. Accordingly, the impact of the second approach, which took increasing life expectancy into account, on SMR was small in Spain, but rather strong in Sweden.

The year 2020 was a leap year. Thus, it was 0.75 days longer than the average year in the time interval 2016 to 2019. To correct for this, the number of observed deaths in the whole year has to be multiplied by (366.25–0.75) / 366.25 = 0.998. This has very little effect on the cumulative SMRs by the end of the year, e.g., the point estimate would change from 0.976 to 0.974 in Germany.

Between country differences in excess mortality are probably due to several factors, and a final assessment may only be possible at the end of the pandemic. However, some reasons for mortality trajectories in Sweden and Spain 2020 have been discussed. Sweden had a higher COVID-19 related death rate than Norway, Finland and Denmark from February to July 2020 [13]. This has been related to the fact that mobility reduction in Sweden was only half as much as in the neighboring countries, and that the government measures were rather lax at the beginning of the pandemic [13]. Others argued that the ability of Swedish nursing homes to protect their inmates was overestimated at the beginning of the pandemic, and that core elements of the Swedish COVID-19 containment strategy, in particular, achieving herd immunity early, have not proven to be correct [14]. High excess mortality in Spain was related to a disproportionate shortfall of nurses and doctors, a lack of diagnostic tests and of protective equipment at the beginning of the pandemic [15]. Others pointed out that travelling from and to Madrid led to the spread of the virus in the province [16]. In general, in persons with COVID-19, obesity, hypertension, diabetes and cardiovascular disease are risk factors for severe courses of COVID-19 and, thus, higher risks of mortality [17–19]. Therefore, the extent of excess mortality may also depend on how prevalent these chronic diseases are across countries.

### Limitations and strengths

A limitation of the study is that the population figures were only available for the middle of the year whereas numbers of death were available on a week-specific basis. Moreover, our analyses do not capture sex-specific differences in excess mortality because sex-specific data on weekly mortality were not available in the Eurostat database.

One strength of the present study is that the demographic development was taken into account in the present study. In Germany, there were 4.8 million people aged 80 and over in 2016 and 5.8 million in 2020, so that more deaths could be expected in 2020 even without COVID-19. Taking into account that we used the population for 31^st^ December 2019 for the whole year 2020, and assuming that the number of people aged 80 and over was larger in the middle of 2020 than half a year earlier, it has to be anticipated that the true SMRs are even smaller. In other studies on excess mortality in Europe, only net numbers of deaths were considered. Another strength compared to other studies is the cumulative consideration of mortality instead of focussing on individual calendar weeks alone. Furthermore, we also considered increased life expectancy what had not been done in other studies on excess mortality due to Covid-19.

## Conclusions

Excess mortality is a reliable indicator of health consequences of the SARS-CoV-2 pandemic for two reasons: first, excess mortality is largely attributable to Covid-19 given that there were no other public health events, and, second, death from any cause does not show ambiguity like death due to or with SARS-Cov-2, sometimes labeled as COVID-19 deaths. However, it is not enough to base this kind of analysis on net numbers of death. As our analyses indicate changes in the age composition of the society, particularly larger numbers of elderly persons, and improvements of life expectancy have to be taken into account to get a proper idea of the health consequences of the pandemic.

A continuation of these analyses to describe the further course of the pandemic is warranted. In particular, long-term studies will show whether excess mortality during the second wave will be followed by submortality in the subsequent months.
